# Two new *Cochlostoma* species from the Durmitor and Komarnica regions of northern Montenegro (Caenogastropoda, Cochlostomatidae)

**DOI:** 10.3897/BDJ.14.e196955

**Published:** 2026-07-27

**Authors:** Menno Schilthuizen, Rob Boomsma, Sofie Faes, Marije Jousma, Janine Buisman, Dirk-H. Lankenau, Sotiris Kountouras, Marlijn van den Broek, Elisabeth M. Hemelaar, Jeroen Noomen, Rick de Vries, Giulia Lopatriello, Antonina Rita Limongi, Simone Maestri, Panche Kamchev, Siebe van Manen, Mohd Zacaery Khalik, Massimo Delledonne, Jovana Đokić, Andrijana Mićanović, Jelena Popović, Iva Njunjić, Enrico Zallot

**Affiliations:** 1 Taxon Expeditions, Leiden, Netherlands Taxon Expeditions Leiden Netherlands; 2 Naturalis Biodiversity Center, Leiden, Netherlands Naturalis Biodiversity Center Leiden Netherlands; 3 Leiden University, Leiden, Netherlands Leiden University Leiden Netherlands; 4 Taxon Foundation, Leiden, Netherlands Taxon Foundation Leiden Netherlands; 5 Rijksuniversiteit Groningen, Groningen, Netherlands Rijksuniversiteit Groningen Groningen Netherlands; 6 University of Verona, Verona (VR), Italy University of Verona Verona (VR) Italy; 7 Faculty of Resource Science and Technology, Universiti Malaysia Sarawak, Kota Samarahan, Malaysia Faculty of Resource Science and Technology, Universiti Malaysia Sarawak Kota Samarahan Malaysia; 8 Montenegrin Ecologists Society, Podgorica, Montenegro Montenegrin Ecologists Society Podgorica Montenegro; 9 Muséum National d'Histoire Naturelle, Paris, France Muséum National d'Histoire Naturelle Paris France; 10 Via per Ligont 1, Dardago di Budoia, 33070, Italy Via per Ligont 1 Dardago di Budoia, 33070 Italy

**Keywords:** anatomy, Balkans, citizen science, conchology, DNA barcoding, Gastropoda, Mollusca, *
Turritus
*

## Abstract

**Background:**

Slow-migrating gastropods are key indicators of habitat continuity, i.e. areas of relatively long-term ecological stability. The gastropod genus *Cochlostoma* (Architaenioglossa, Cochlostomatidae) is a common and widespread component of karst communities throughout the countries surrounding the Mediterranean. Recent work has taken into account genetic and reproductive-anatomical diversity and has revealed that the uniform shell morphology hides surprisingly rich and deep divergences.

**New information:**

Here, we describe two species of the subgenus *Turritus*, C. (Turritus) dobrido sp. nov. and C. (Turritus) komarnica sp. nov. They were collected, studied and described as part of citizen-science expeditions to the threatened Komarnica Canyon and to Durmitor National Park, two adjoining regions in Montenegro. The new species were found sympatrically and, in some cases, syntopically with *Cochlostoma scalariniforme* (A. J. Wagner, 1906), as well as with each other.

## Introduction

The genus *Cochlostoma* Jan, 1830 is a common, well-known and widespread component of the malacofauna of limestone rocks throughout the countries surrounding the Mediterranean. The animals have small to medium-sized conical shells that have been the subject of many older taxonomic studies (e.g. Westerlund 1885, Wagner 1897, Schütt 1977), which relied on shell shape, pigmentation and conchological details to delineate species and other infrageneric taxa. Recent work (Zallot et al. 2015, Zallot et al. 2018, Zallot et al. 2021), however, has revealed that the taxonomic usefulness of shell characters is limited and, instead, the female anatomy paired with DNA data, reveal a rich and clearer picture of the intrageneric and species-level taxonomy.

Zallot et al. (2015), using evidence from conchology, anatomy and DNA, distinguished eight subgenera within the genus. Three of these, *Lovcenia* Zallot et al., 2015, *Clessiniella* Zallot et al., 2015 and *Turritus* Westerlund, 1883, have been recently revised (Zallot et al. 2018, Zallot et al. 2021, Zallot et al. 2024). The latter subgenus consists of at least 37, partly cryptic, species distributed throughout the Italian and Balkan peninsulas, the eastern and western parts of the Alps, the southern coastal mountains of France and Spain, Sardinia and Sicily. One species known from the Algerian coast may also belong to *Turritus*. While some morphological traits are diagnostic to separate *Turritus* from some of the other subgenera, no single trait is sufficiently unique to morphologically define *Turritus*. Nonetheless, molecular phylogenetics show that it is a strongly supported monophyletic group, subdivided into two molecularly well-defined clades, A and B (Zallot et al. 2015).

In addition to delineating 37 species in *Turritus*, Zallot et al. (2024) also highlighted the existence of populations and forms within *Turritus* that require further study before their species identity could be ascertained. This was either because of missing anatomical and/or genetic data or because of contradictory evidence from different character systems. Two of these forms, termed 'NFS142' and 'NFS143', were populations belonging to Clade B, which had been found at high elevations in northern Montenegro. However, because of insufficient materials, Zallot et al. (2024) refrained from formally describing them until further material became available.

**Figure 1.**
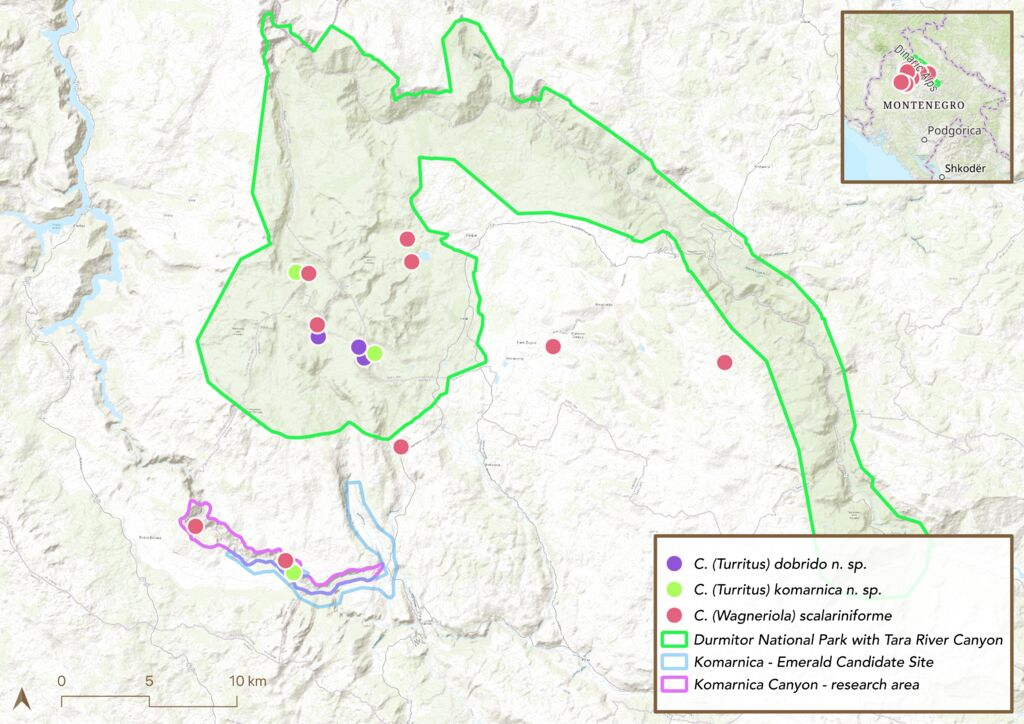
Distribution map of all genetically confirmed specimens of *C. dobrido*, *C. komarnica* and *C. scalariniforme* in the Durmitor and Komarnica area (in north-western Montenegro). "Emerald candidate site" refers to site ME000000P under the Bern Convention (Popović et al. 2024). Some occurrences that were very close together were shifted slightly to prevent occlusion. Map by J. Popović.

Between June 2018 and July 2025, we sampled extensively in the contiguous areas of the Komarnica Canyon and Durmitor National Park (Fig. 1). Komarnica Canyon is currently threatened by a planned hydropower dam and reservoir (Vujović et al. 2023, Popović et al. 2024) and is, therefore, the focus for structured biodiversity surveys in an attempt to build a science-based case for its conservation. During these sampling events, we obtained numerous *Cochlostoma* specimens, including 'NFS142', 'NFS143' and previously described species. The work was carried out by mixed teams of local and international laypeople and taxonomists as part of a ‘taxon expedition’ (Freitag et al. 2018), in which ‘taxonomy tourists’ are guided by biodiversity experts through the entire process of species discovery and description. As part of the field course programmes, we took this opportunity to produce detailed descriptions of 'NFS142' and 'NFS143', using conchology, anatomy and DNA sequence data in these populations. We here formally describe two new species, and provide sufficient data for other, similar and possibly conspecific populations for future comparisons.

## Materials and methods

### Fieldwork

Between June 2018 and July 2025, we sampled *Cochlostoma* specimens at multiple sites in the Komarnica Canyon and Durmitor National Park. Here, *Cochlostoma* populations often are dense and common on Triassic or Cretaceous limestone (Đurović 2009). During dry weather, specimens were found in crevices in the rocks and hidden under mosses and at plant roots. Empty shells were sieved from debris underneath vertical limestone rocks, retrieved either by direct inspection of the sieved material or by flotation, then dried and stored in plastic or glass vials. During wet weather, snails were found actively foraging on the rocks and in moss and lichen. Specimens were collected in the field in clean, empty plastic bags or vials and transported to the field lab. Here, adult individuals were relaxed overnight in water without air bubbles and then transferred into 70% ethanol. Tissue samples of ca. 1 mm^3^ were taken by removing the apical 2-3 whorls of the shell with the soft body inside and preserved in 96% ethanol for further molecular work. All sampled specimens are stored in the Taxon Expeditions Collection (collection coden TXEX), Leiden, the collection of Naturalis Biodiversity Center, Leiden (collection coden RMNH), the National Museum of Natural History of Montenegro in Podgorica (collection coden NHMM) and the private collection of Enrico Zallot, Dardago di Budoia, Italy (abbreviated with EZ). Localities in the Durmitor and Komarnica region were given locality codes of the form, TxExDU####, which are used throughout this paper. Details of these localities are given in Table 1.

### Conchology

**Figure 2.**
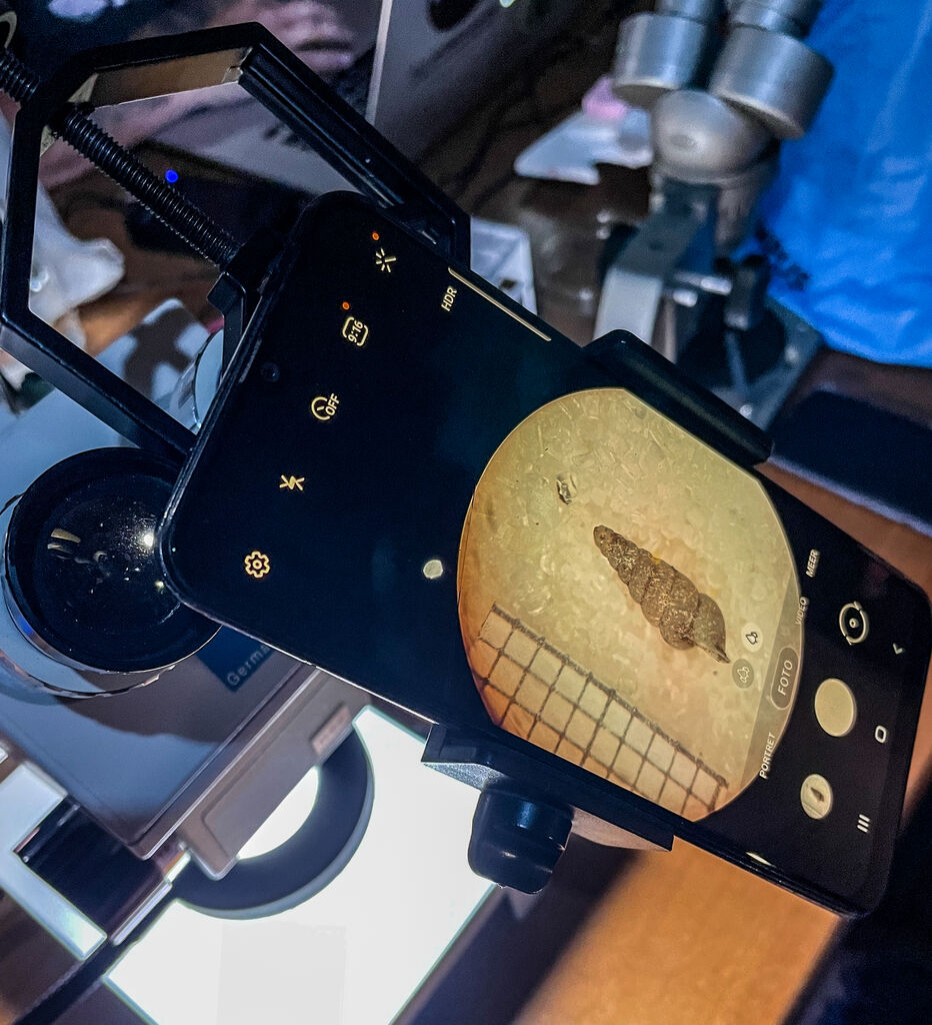
Improvised photo set-up in the field, using a smartphone adapter on the eyepiece of a small dissection microscope (photo: D.-H. Lankenau).

For studying shell morphology, we photographed shells with a smartphone through the eyepiece of a Zeiss GSZ dissection microscope in apertural and lateral view, always taking care that the angle of view was perpendicular to the columella (Fig. 2). A piece of graph paper was photographed along with the shell for scale. Using the programme ImageJ (Rasband 2016), these images were then used to extract shell measurements as described in Zallot et al. (2024), namely: shell height; height of the body whorl; width of the body whorl; width of the suture of the body whorl; width of the penultimate whorl; width of the penultimate suture; inclination of the ribs on the body whorl in relation to the orthogonal line to the main axis; inclination of the ribs on the 4^th^ whorl measured from the aperture in relation to the orthogonal line to the main axis; inclination of the aperture lip in relation to the main axis in lateral view; ribs per millimetre measured along the suture of the body whorl; and ribs per millimetre measured along the basal suture of the 4^th^ whorl, measured from the aperture. The same shells were used to score the following qualitative characters: umbilicus (closed or open), columellar lobe (flat or indented), shell colour (spotted or not), ribbing (weak or strong). All measurements were taken by author M.J.

### Reproductive anatomy

To study the reproductive anatomy, we carefully chipped away the shells of females preserved in 70% ethanol and used the soft bodies to dissect the female reproductive system, the layout of which was photographed with a smartphone through the eyepiece of a Zeiss GSZ dissection microscope and also traced for schematic drawings. The terminology as well as the orientations (e.g. ventral, dorsal) follow Zallot et al. (2015).

### Molecular work and phylogenetics

We performed molecular analyses on representative specimens from the Durmitor and Komarnica area that could not be assigned conchologically to any species with certainty. (In the northern part of the region, in the bottom of the Tara Canyon, localities TxExDU0037, TxExDU0040 and TxExDU0041, we also found Cochlostoma (Cochlostoma) septemspirale (Razoumowski, 1789), which is easily recognisable by its open umbilicus). Tissue samples (prepared as described above) were analysed genetically, either with a portable field lab (Menegon et al. 2017, Maestri et al. 2019) or later in the permanent lab in Leiden.

For the portable field lab, the protocols conformed to the following procedures. DNA extraction from snail tissue was performed with the DNeasy Blood & Tissue Kit (Qiagen), following the recommended protocol. The extracted DNA served as a template for PCR amplification, specifically targeting cytochrome c oxidase subunit I (COI) or 16S mitochondrial DNA (16S mtDNA) barcoding regions, using the primers reported in Folmer et al. (1994) and Palumbi and Kessing (1991), respectively, tailed at the 5’ end with Oxford Nanopore adapters. Subsequently, the amplicons underwent electrophoresis on a 0.8% agarose gel to confirm fragment size and assess the absence of contamination. Successful amplicons were uniquely tagged with sample-specific barcodes using the PCR-Barcoding Kit (Oxford Nanopore Technologies). To ensure purity, AMPureX magnetic beads were applied at a 0.5x ratio to remove contaminants. Amplicon quantification was performed using a Qubit fluorometer. DNA libraries were prepared from the pool of amplicons with the Ligation Sequencing Kit V.8 (2019) and V.14 (2023) (Oxford Nanopore Technologies), following the recommended protocols. Sequencing was carried out using the MinION device (Oxford Nanopore Technologies) with an R9.4 (2019) and an R10.4.1 (2023) flow cell, with data acquisition managed by MiniKNOW software, v.1.6.11 (2019) and 23.04.3 (2023) (Oxford Nanopore Technologies). Following sequencing, the POD5 files generated by the MinION device underwent base-calling using the ONT Preprocessing Pipeline, powered by Guppy 6.5.7, to produce fastq files. These fastq files served as input for the ONTrack2 pipeline (Maestri et al. 2019), which generated a consensus sequence.

For the Leiden lab, the protocols followed were similar, but differed as follows. For some samples, DNA was extracted with the E.Z.N.A Mollusc DNA Kit (Omega Bio-tek), following the manufacturer's protocol. PCR-primers were not tailed with adapters. Successful amplicons were cleaned with the ExoSAP-IT protocol (Thermo Fischer Scientific) and sequenced in both directions at the Sanger-sequencing service of Eurofins Scientific and Macrogen Europe. After sequencing, primer sequences were removed and unreliable bases were replaced with an ambiguity nucleotide ("N"). The sequences were deposited in GenBank under accession numbers PZ298044-PZ298053 and PZ298552-PZ298571.

We aligned all (COI and 16S) concatenated sequences in Geneious Prime v.2020.0.4 using the MUSCLE algorithm (Edgar 2004) and default settings. The alignment was then subjected to a Maximum-Likelihood analysis using PhyML as incorporated in Geneious Prime (Guindon et al. 2010), with a GTR substitution model, fixed proportion of invariable sites, four substitution rate categories, estimated gamma distribution and 100 bootstrap replicates. Trees were displayed and manipulated in FigTree v.1.4.2. Trees were rooted with C. (Wagneriola) scalariniforme.

## Taxon treatments

### Cochlostoma (Turritus) dobrido

Schilthuizen & Zallot sp. nov.

1589F92D-40CB-5709-AD38-DE8ED8C5F4D8

2EA7BE4A-A100-4510-B7B4-8CA24136E4DB

synonyms: Cochlostoma (Turritus) “NFS143” in Zallot et al. (2024).

#### Materials

- **Type status:** Holotype. **Occurrence:** recordedBy: M. Schilthuizen; individualCount: 1; sex: female; lifeStage: adult; occurrenceID: 7BFDAF5B-F531-5EA5-9B51-DE5622433412; **Taxon:** kingdom: Animalia; phylum: Mollusca; class: Gastropoda; order: Architaenioglossa; family: Cochlostomatidae; genus: Cochlostoma; subgenus: Turritus; specificEpithet: dobrido; taxonRank: species; scientificNameAuthorship: Schilthuizen & Zallot, 2026; nomenclaturalCode: ICZN; **Location:** country: Montenegro; locality: Durmitor National Park: Dobri Do; verbatimElevation: 1850 m; decimalLatitude: 43.09885; decimalLongitude: 19.04812; geodeticDatum: WGS84; **Event:** year: 2024; month: 7; day: 14; fieldNumber: TxExDU0181; **Record Level:** type: PhysicalObject; institutionID: NHMM; collectionID: NHMM; institutionCode: NHMM; basisOfRecord: PreservedSpecimen
- **Type status:** Other material. **Occurrence:** recordedBy: M. Schilthuizen; individualCount: 78; lifeStage: adult; occurrenceID: BD7BC8D2-A910-5D75-83E8-CF95B16903B7; **Taxon:** kingdom: Animalia; phylum: Mollusca; class: Gastropoda; order: Architaenioglossa; family: Cochlostomatidae; genus: Cochlostoma; subgenus: Turritus; specificEpithet: dobrido; taxonRank: species; scientificNameAuthorship: Schilthuizen & Zallot, 2026; nomenclaturalCode: ICZN; **Location:** country: Montenegro; locality: Durmitor National Park: Dobri Do; verbatimElevation: 1850 m; decimalLatitude: 43.09885; decimalLongitude: 19.04812; geodeticDatum: WGS84; **Event:** year: 2024; month: 7; day: 14; fieldNumber: TxExDU0181; **Record Level:** type: PhysicalObject; institutionID: TXEX; collectionID: TXEX; institutionCode: TXEX; basisOfRecord: PreservedSpecimen

#### Description

##### Shell

**Figure 3.**
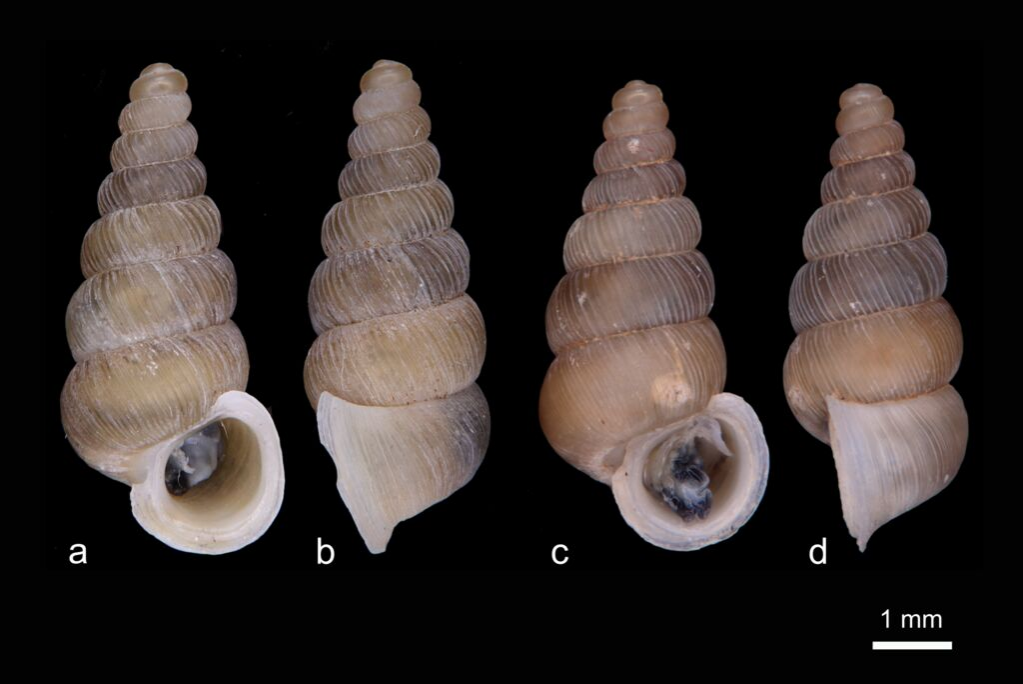
Shell shapes of *Cochlostoma komarnica* sp. nov. from Škrčko Jezero, locality TXEXDU0124 (a, apertural view; b, lateral view) and *C. dobrido* sp. nov. from Dobri Do, locality TXEXDU0181 (c, apertural view; d, lateral view). Photos by E. Zallot.

Shell pale yellowish-grey, without any orange spots; 7.3-8.0 whorls. On the protoconch, riblets appear after 1.40-2.20 whorls. Shell height 6.33-7.10 mm (mean 6.78, s.d. 0.247). Shell width 2.62-2.83 mm (mean 2.71, s.d. 0.066). Shell index (shell height / body whorl width) 2.37-2.58. Height of the body whorl: 1.43-1.62 mm (mean 1.49, s.d. 0.066). Width of the suture between the body whorl and the penultimate whorl: 2.15-2.36 mm (2.22, s.d. 0.071). Width of the penultimate whorl: 2.32-2.55 mm (mean 2.41, s.d. 0.073). Width of the suture between the penultimate whorl and the teleoconch: 1.77-1.96 mm (mean 1.87, s.d. 0.061). Inclination of the ribs on the body whorl relative to the orthogonal line to the main axis: 17-28° (mean 21.5, s.d. 3.41). Inclination of the ribs on the 4^th^ whorl (counted from the aperture) relative to the orthogonal line to the main axis: 17-25° (mean 21.1, s.d. 2.60). Inclination of the aperture relative to the orthogonal line to the main axis in lateral view: 7-18° (mean 11.3, s.d. 3.77). Rib density on the body whorl (measured just basal of the suture between the body whorl and the penultimate whorl: 8-16 per mm (mean 10.9, s.d. 2.96). Rib density basal on the fourth whorl (counted from the aperture): 7-14 per mm (mean 10.9, s.d. 1.96). The umbilicus is hidden. Columellar lobe indented. Radial ribs weak. An overview of the shell is in Fig. 3.

##### Anatomy

**Figure 4.**
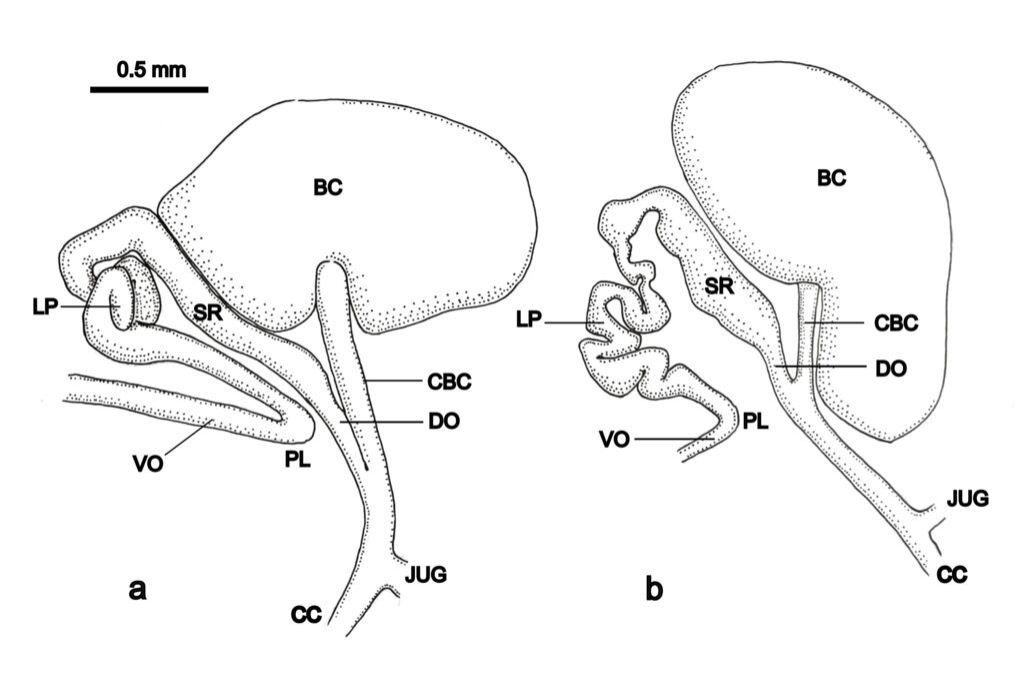
Diagnostic part of the female reproductive system in *Cochlostoma komarnica* sp. nov. (a) and *C. dobrido* sp. nov. (b). CBC: pedunculus of the bursa copulatrix; CC: copulatory duct; DO: distal oviduct; JUG: channel of the uterine gland; LP: loops of the visceral oviduct; PL: proximal loop; SR: seminal receptacle; VO: visceral oviduct. Drawings by M. Schilthuizen.

(Abbreviations refer to Fig. 4) The pendunculus (CDC) connects ventrally to the bursa copulatrix (BC). The seminal receptacle (SR) is relatively short and club-shaped, with the apex positioned at the middle of the body (i.e. the seminal receptacle does not reach the dorsal side). The visceral oviduct shows several tight loops (LP) clustered just ventral of the apex of the seminal receptacle. The junction to the channel of the uterine gland (JUG) is situated relatively far (0.4-0.5 mm) from the connection between the pedunculus of the bursa and the distal oviduct (DO).

##### DNA barcode

A representative Cytochrome Oxidase I DNA barcode from the type locality:

5'AAACAAATGTTGATATAATACAGGATCACCCCCCCAGCAGGATCAAAAAAAAGAAGTATTAAAATTACGATCTGTTAATAATATAGTAATAGCTCCTGCTAAAACAGGAAGTGATAATAATAAAAGAATAGCTGTAATTTTAACAGATCAAACAAATAAAGGTAAACGTTCAAAAGACATTCCCTGTCAACGTATATTAATAATTGTAGTAATAAAATTAACAGCTCCTAAAATAGAAGAGGCTCCTGCTAAATGTAAAGAAAAAATAGCAAGATCTACAGATCCACCAGCGTGAGCAAGATTTCCTGATAGAGGGGGATAAACTGTTCAACCCGTTCCTGCACCTCTTTCAACGGCCGCAGAAGATAATAAAAGCAACAACGAAGGAGGTAATAATCAAAACTTATATTATTTAAACGAGGGAAAGCTATATCAGGCGCTCCTAACATTAAAGGAACTAATCAATTTCCAAAGCCCCCAATTATTATAGGTATAACAAAGAAAAAATCATTACAAAAAGCATGAGCAGTTACAATAACATTATATAATTGGTCATCTCCTAATAAAGCGCCTGGTTGCCCTAATTCAGCTCGAATTAATAATCTTAAACCAGTTCCAACTAAACCCGATCACATTCCTAAAATAATATATAAAGTA3'

#### Diagnosis

The phylogenetic analysis supports monophyly for *C. dobrido*. Amongst the four *Cochlostoma* species that occur in this part of Montenegro, *C. dobrido* is conchologically recognised by the densely, weakly ribbed, relatively squat shell (shell index 2.37-2.58) that has a closed umbilicus and no orange spots. *C. scalariniforme* and *C. septemspirale* have orange spots; *C. septemspirale* has an open umbilicus; *C. komarnica* is, on average, more slender (shell index 2.44-2.65). Anatomically, *C. dobrido* is easily distinguishable from *C. septemspirale* and *C. scalariniforme*, as these latter two possess a receptaculum seminis that is bent under a 180 degree angle with respect to the distal oviduct, whereas, in *C. dobrido* and *C. komarnica*, the receptaculum seminis is in line with the distal oviduct. We refer to Zallot et al. (2024) for details on the characteristics of other, not closely-related species occurring in Montenegro, i.e. *C. pallgergelyi* Zallot et al., 2024 and *C. arnautorum* (Wagner, 1906).

#### Etymology

The species is named after the locus typicus, highland plain Dobri Do in Durmitor National Park, here used as a noun in apposition. The name was chosen by vote on the final night of the 2023 Taxon Expedition to Durmitor National Park.

#### Distribution

So far, the species has been reliably recorded (i.e. confirmed by both anatomy and DNA) from three localities in the Dobri Do highland plain. It may be more widespread.

#### Ecology

**Figure 5.**
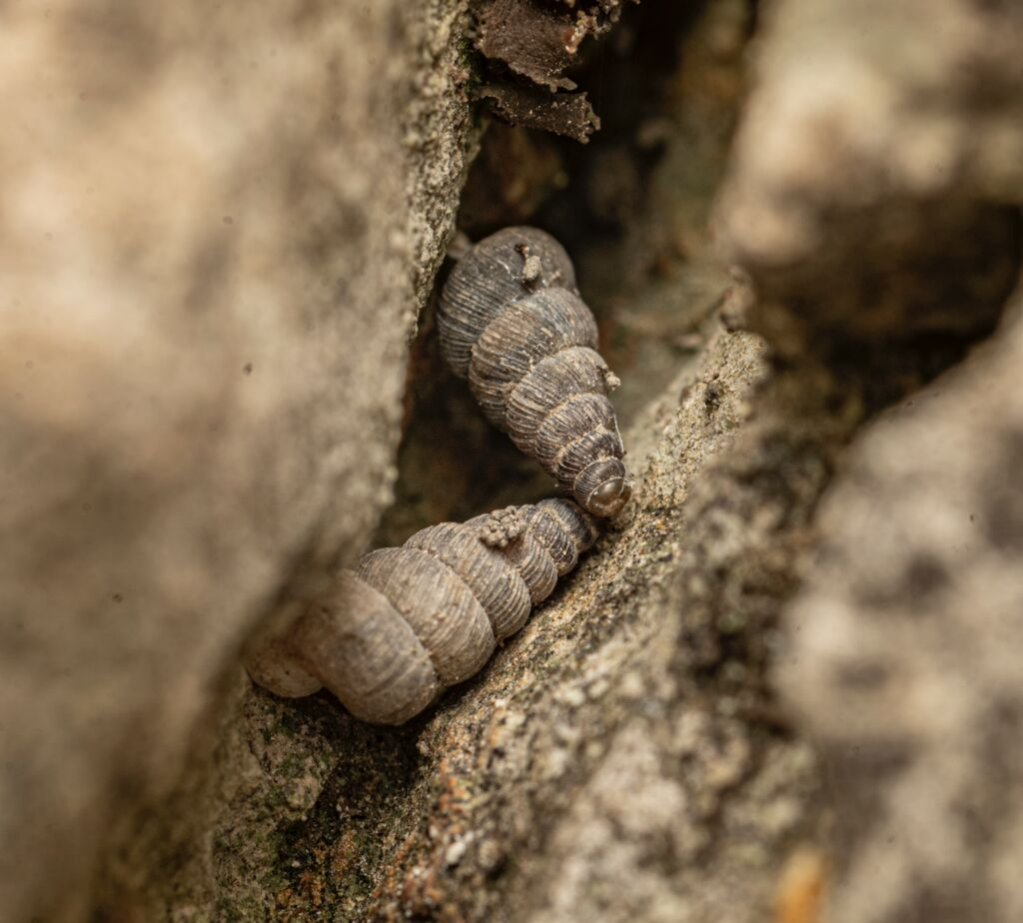
Two live individuals of *Cochlostoma dobrido* sp. nov., during estivation at locality TXEXDU0145. Photo by S. Kountouras.

The species was found in high densities on limestone cliffs between 1680 and 2080 m elevation, sometimes syntopic with *C. komarnica*. The fieldwork was conducted during dry periods in July and most living individuals were found hiding in crevices and beneath layers of moss (Fig. 5).

#### Conservation

Based on the currently known localities (at elevations between 1680 and 2080 m), *C. dobrido* might be endemic to the Dobri Do highland plain and may be adapted to highland environmental conditions. This means its populations may suffer if climate change continues to shift montane environmental zones upward.

### Cochlostoma (Turritus) komarnica

Schilthuizen & Zallot sp. nov.

347DF93C-A36F-5CCC-B0AD-D3FF5880EB76

C156D929-4DF2-4E58-BC32-1F8D52D2B978

Synonyms: Cochlostoma (Turritus) “NFS142” in Zallot et al. (2024).

#### Materials

- **Type status:** Holotype. **Occurrence:** recordedBy: Taxon Expedition participants; individualCount: 1; sex: male; lifeStage: adult; occurrenceID: C0F2591D-3D58-5249-AE76-75D24C05C28E; **Taxon:** kingdom: Animalia; phylum: Mollusca; class: Gastropoda; order: Architaenioglossa; family: Cochlostomatidae; genus: Cochlostoma; subgenus: Turritus; specificEpithet: komarnica; scientificNameAuthorship: Schilthuizen & Zallot, 2026; nomenclaturalCode: ICZN; **Location:** country: Montenegro; locality: Komarnica Canyon: left bank of the river; verbatimElevation: 730 m; decimalLatitude: 42.98222; decimalLongitude: 19.00611; geodeticDatum: WGS84; **Event:** year: 2024; month: 7; day: 25; fieldNumber: TxExDU0193; **Record Level:** type: PhysicalObject; institutionID: NHMM; institutionCode: NHMM; ownerInstitutionCode: NHMM; basisOfRecord: PreservedSpecimen
- **Type status:** Other material. **Occurrence:** catalogNumber: TXEX.MOL.00152; recordedBy: Taxon Expedition participants; individualCount: 175; lifeStage: adult; occurrenceID: 91A7DFA7-2A25-574C-AFC0-5355A16F1F33; **Taxon:** kingdom: Animalia; phylum: Mollusca; class: Gastropoda; order: Architaenioglossa; family: Cochlostomatidae; genus: Cochlostoma; subgenus: Turritus; specificEpithet: komarnica; scientificNameAuthorship: Schilthuizen & Zallot, 2026; nomenclaturalCode: ICZN; **Location:** country: Montenegro; locality: Komarnica Canyon: left bank of the river; verbatimElevation: 730 m; decimalLatitude: 42.98222; decimalLongitude: 19.00611; geodeticDatum: WGS84; **Event:** year: 2024; month: 7; day: 25; fieldNumber: TxExDU0193; **Record Level:** type: PhysicalObject; institutionID: TXEX; institutionCode: TXEX; ownerInstitutionCode: TXEX; basisOfRecord: PreservedSpecimen

#### Description

##### Shell

Shell pale yellowish-grey, without any orange spots; 7.3-8.4 whorls. On the protoconch, riblets appear after 1.2-1.8 whorls. Shell height 5.12-5.79 mm (mean 5.46, s.d. 0.221). Shell width 1.97-2.35 mm (mean 2.14, s.d. 0.109). Shell index (shell height / body whorl width) 2.44-2.65. Height of the body whorl: 1.09-1.25 mm (mean 1.17, s.d. 0.051). Width of the suture between the body whorl and the penultimate whorl: 1.67-1.99 mm (mean 1.79, s.d. 0.96). Width of the penultimate whorl: 1.81-2.10 mm (mean 1.94, s.d. 0.087). Width of the suture between the penultimate whorl and the teleoconch: 1.43-1.60 mm (mean 1.51, s.d. 0.055). Inclination of the ribs on the body whorl relative to the orthogonal line to the main axis: 24-31° (mean 27, s.d. 2.49). Inclination of the ribs on the 4^th^ whorl (counted from the aperture) relative to the orthogonal line to the main axis: 20-29° (mean 23.2, s.d. 3.12). Inclination of the aperture relative to the orthogonal line to the main axis in lateral view: 13-23° (mean 18.8, s.d. 2.69). Rib density on the body whorl (measured just basal of the suture between the body whorl and the penultimate whorl: 6-9 per mm (mean 7.56, s.d. 1.33). Rib density on the fourth whorl (counted from the aperture): 10-13 per mm (mean 11.1, s.d. 0.99). The umbilicus is hidden. Columellar lobe indented. Radial ribs moderately strong. An overview of the shell is in Fig. 3.

##### Anatomy

Female reproductive system. (Abbreviations refer to Fig. 4). Ventral connection of pedunculus (CDC) to bursa copulatrix (BC). Relatively short, club-shaped, seminal receptacle (SR) with apex positioned at middle of body (seminal receptacle not reaching dorsal side, but not confined to ventral side). Ventral of the apex of the seminal receptable, the visceral oviduct (VO) bends ventrally and then shows a few tight loops (LP). Junction of uterus gland (JUG) far from connection between the pedunculus of bursa copulatrix and distal oviduct (DO).

##### DNA barcode

A representative Cytochrome Oxidase I DNA barcode from the type locality:

5'AAACAAATGTTGATATAATACAGGGTCCCCTCCTCCAGCTGGATCAAAAAAGTAGTATTAAAGTTACGATCTGTTAATAATATAGTGATAGCTCCTGCTAAAACAGGAAGTGATAATAATAAAAGAATAGCTGTAATTTTAACAGATCAAACAAATAAAGGTAGACGTTCAAAAGATATTCCATGTCAACGTATATTAATAATCGTAGTAATAAAATTAACAGCACCTAAAATAGAAGAAGCTCCTGCTAAATGTAAAGAGAAAATAGCAAGATCTACAGATCCCCCCAGCATGGGCAAGATTCCCTGATAAAGGAGGATAAACTGTCCAACCCGTTCCTGCACCTCTTTCAACAGCTGCAGAAGATAATAAAAGTAATAACGAAGGTGGTAATAACCAAAGCTTATATTATTTAAACGAGGAAAAGCTATATCAGGAGCCCCTAATATTAAAGGGACCAACCAATTTCCAAAACCTCCAATCATTATAGGTATAACAAAAAAAAAAATTATTACAAAAAGCATGAGCAGTTACAATAACATTATATAACTGGTCATCTCCTAATAAAGCACCTGGTTGTCCTAGCTCAGCTCGAATTAATAATCTCAAACCTGTTCCGACTAACCCCGATCATATACCTAAAATAATATATAAGGTA3'

#### Diagnosis

The phylogenetic analysis supports monophyly for *C. komarnica*. Amongst the four *Cochlostoma* species that occur in this part of Montenegro, *C. komarnica* may be recognised conchologically by the moderately strongly ribbed, relatively slender shell (shell index 2.44-2.65) that has a closed umbilicus and no orange spots. *C. scalariniforme* and *C. septemspirale* have orange spots; *C. septemspirale* has an open umbilicus; *C. dobrido* is, on average, more slender (shell index 2.37-2.58). Anatomically, *C. komarnica* is easily distinguishable from *C. septemspirale* and *C. scalariniforme*, as these latter two possess a receptaculum seminis that is bent under a 180 degree angle with respect to the distal oviduct, whereas, in *C. dobrido* and *C. komarnica*, the receptaculum seminis is in line with the distal oviduct. As Zallot et al. (2024) observed (using the term "NFS142" for the entity we now describe as *C. komarnica*), there is a genetically and morphologically close relationship with *C. hallgassi* Zallot et al., 2024, which occurs north of Naples and is, thus, separated geographically by the Adriatic Sea and the Italian peninsula. Conchologically, the two species differ chiefly by the pattern of two spiral rows of orange dots on the shell of *C. hallgassi*. We refer to Zallot et al. (2024) for details on the characteristics of other, not closely-related species occurring in Montenegro, i.e. *C. pallgergelyi* Zallot et al., 2024 and *C. arnautorum* (Wagner, 1906).

#### Etymology

The species is named after the locus typicus, Komarnica Canyon. The name *komarnica* is used as a noun in apposition.

#### Distribution

The species appears to be endemic to the Durmitor National Park and the adjacent Komarnica Canyon and probably also occurs in the narrow area in between. Its elevational range is quite broad, having been recorded from 730 to 1735 m elevation.

#### Ecology

The species was found in high densities on limestone cliffs between 730 and 1735 m elevation, sometimes syntopic with *C. scalariniforme* or *C. dobrido*.

#### Conservation

While the populations at higher elevations are within the Durmitor National Park, those at lower elevation occur inside the continuity habitats of the Komarnica Canyon, which is threatened by a planned hydropower dam. Should this project be realised, the type locality will flood and this population will perish.

## Discussion

**Figure 6.**
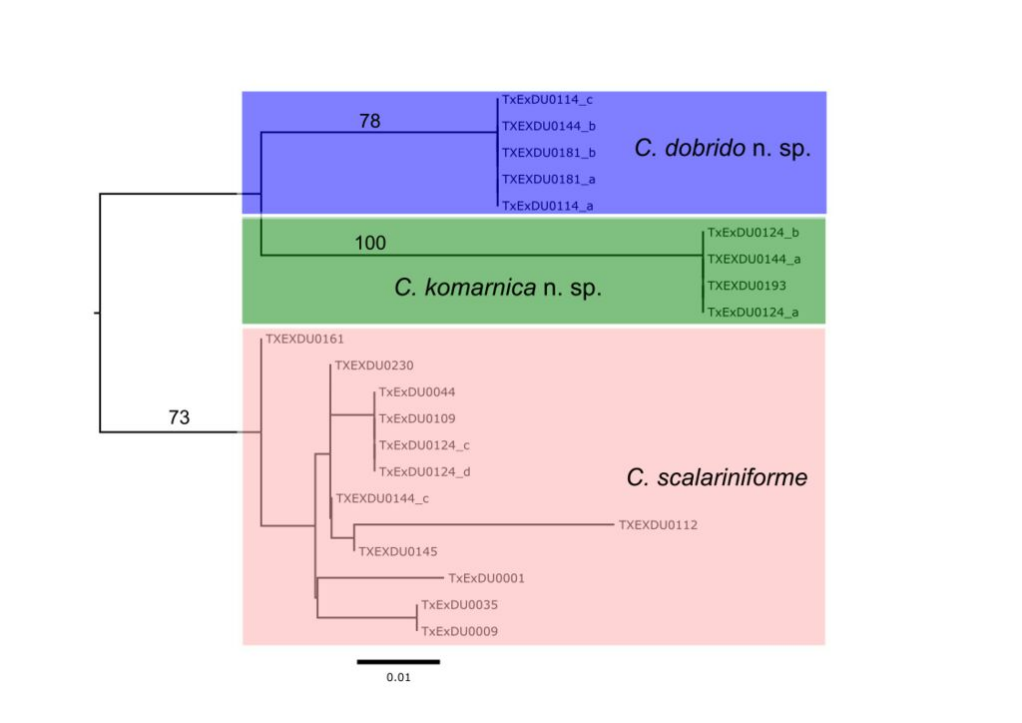
A Maximum-Likelihood-based phylogeny reconstruction for concatenated COI and 16S sequences for *Cochlostoma* individuals from Durmitor and Komarnica. The tip labels refer to localities presented in Table 1. The node labels are selected bootstrap percentages. Figure by M. Schilthuizen.

Our molecular analysis showed that C. (Turritus) dobrido and C. (Turritus) komarnica are genetically (based on two partial mtDNA loci) quite distant (ca. 8%) from each other and are also genetically well-separated from C. (Wagneriola) scalariniforme (Fig. 6). Conchologically, however, the separation from the latter is difficult, which is why we relied on DNA sequence data for confirming the identity of any specimen found in the study area. Anatomically, *C. dobrido* and *C. komarnica* are quite similar and much of the difference in Fig. 4 may be due to individual, not species-level variability. Nonetheless, as the diagnoses highlight, the two new species can be distinguished from each other and from the other *Cochlostoma* species occurring in the area by considering a combination of conchological, genetic and anatomical features.

In Fig. 1, we present the localities of all thus far genetically confirmed individuals. Still, the challenges in identifying individuals, combined with the fact that two species might occur in mixed syntopic populations, suggest that reliable distribution maps for each of the species will need to await further research. It is, therefore, also possible that more new species may turn up. The area in which Durmitor Mountain and Komarnica Canyon are located is a geologically and ecologically complex region with many canyons, plains, gorges and mountains and many microhabitats across a wide elevational range. Consequently, local endemism in these snails may be even more widespread. This is another reason why the construction of the Komarnica Dam and the subsequent flooding of the biodiversity-rich Komarnica Canyon (the type locality of *C. komarnica*) should be prevented (Vujović et al. 2023) and why its inclusion in the Durmitor UNESCO World Heritage Site should be persistently advocated.

## Supplementary Material

XML Treatment for Cochlostoma (Turritus) dobrido

XML Treatment for Cochlostoma (Turritus) komarnica

## Figures and Tables

**Table 1.** Locality codes in the Komarnica and Durmitor region where *Cochlostoma* specimens were sampled.

locality code	locality	collection date	latitude (decimal degrees)	longitude (decimal degrees)	elevation (m)	collectors
TxExDU0001	Crno Jezero, north-facing rocky slope in spruce forest	16 June 2018	43.14327	19.08379	1506	M. Schilthuizen & I. Njunjić
TxExDU0009	Sinjajevina, Šaranci, Zakrajci/Pometenici, Sniježnica Pećina	20 June 2018	43.09502	19.30472	1560	M. Schilthuizen & I. Njunjić
TxExDU0035	Pećina u Barama Žugića, cave entrance	12 July 2018	43.10135	19.18430	1377	Taxon Expeditions participants
TxExDU0037	Tara Canyon, left river bank, Ljutica spring, under rocks	13 July 2018	43.13828	19.30112	837	Taxon Expeditions participants
TxExDU0040	Tara Canyon, south-facing rocky slope by right-hand riverside	13 July 2018	43.18136	19.24084	794	Taxon Expeditions participants
TxExDU0041	Tara Canyon, north-facing rocky slope by left river bank	13 July 2018	43.21888	19.17588	783	Taxon Expeditions participants
TxExDU0044	Škrčko Jezero, boulders and cliffs around the mountain hut	16 July 2018	43.13605	19.01185	1735	Taxon Expeditions participants
TxExDU0109	Žlijeb, rocky gorge	3 July 2019	43.15486	19.08038	1560	M. Schilthuizen, I. Njunjić & T. Mitrović
TxExDU0112	Arapova Pećina, cave entrance	6 July 2019	43.04809	19.07934	1564	M. Schilthuizen, I. Njunjić & T. Mitrović
TxExDU0114	Dobri Do, close to car park for trail to Škrčko Jezero	6 July 2019	43.10365	19.01956	1669	M. Schilthuizen
TxExDU0124	Škrčko Jezero, boulders and cliffs around the mountain hut	13 July 2019	43.13605	19.01185	1735	Taxon Expeditions participants
TxExDU0144	Sedlo: Pećina u Sedlenoj Gredi, cave entrance	18 July 2023	43.09319	19.05287	2087	Taxon Expeditions participants
TxExDU0145	Dobri Do, close to car park for trail to Škrčko Jezero	18 July 2023	43.10299	19.01976	1680	Taxon Expeditions participants
TxExDU0161	Komarnica Canyon: Dragaljica Cave near Duži, cave entrance	27 July 2023	42.98746	19.00524	965	M. Schilthuizen, I. Njunjić & S. Kountouras
TxExDU0181	Dobri Do, close to parking place at Sedlo	15 July 2024	43.09885	19.04812	1850	M. Schilthuizen
TxExDU0193	Komarnica Canyon, left bank of the river	25 July 2024	42.98222	19.00611	730	Taxon Expeditions participants
TxExDU0230	Komarnica Canyon, left bank, Plot E	7 July 2025	43.00480	18.93688	699	S. Faes & M. Schilthuizen c.s.
